# NR5A1-related 46,XY partial gonadal dysgenesis: A case report and literature review

**DOI:** 10.1097/MD.0000000000036725

**Published:** 2023-12-29

**Authors:** Xianzhen Wei, Shan Li, Yu He

**Affiliations:** a Department of Clinical Laboratory, The First Affiliated Hospital of Guangxi Medical University; Key Laboratory of Clinical Laboratory Medicine of Guangxi Department of Education, Nanning city, Guangxi, China.

**Keywords:** 46,XY DSD, disorders of sex development, gonadal dysgenesis, NR5A1

## Abstract

**Rationale:**

Disorders/differences of sex development (DSD) include a diverse group of congenital conditions in which the development of chromosomal, gonadal, or anatomical sex is discordant. It involves several variant genes, and one of them is NR5A1. NR5A1 encodes a signal transduction regulator in the hypothalamic-pituitary-gonadal and hypothalamic-pituitary-adrenal pathway, and pathogenic mutation in this gene is a cause of 46,XY DSD.

**Patient concerns:**

A 12-year-old individual raised as a girl was admitted to the hospital due to hirsutism and a deep voice that began at 11 years old. The individual exhibited testicular hypoplasia, clitoral hypertrophy, and female external genitalia.

**Diagnoses:**

The patient was diagnosed 46,XY partial gonadal dysgenesis. The cytogenetics revealed a 46,XY karyotype and DNA sequencing shown a variant in NR5A1. Pelvic magnetic resonance imaging showed absence of uterus and ovaries. The abdominopelvic ultrasound revealed bilateral testicle in bilateral groin. Pathology confirmed testes dysgenesis.

**Interventions:**

The patient underwent bilateral orchiectomy at age 12 years and was given a feminizing hormonal treatment of 0.5 mg/day of estradiol valerate tablets.

**Outcomes:**

The patient recovered well after surgery and hormonal treatment and had a regression in hirsutism and clitoromegaly.

**Lessons:**

46,XY DSD is a rare disease that the development of chromosomal, gonadal, or anatomical sex is discordant, when diagnosed 46,XY DSD, the identification of an NR5A1 variant should be considered.

## 1. Introduction

Disorders/differences of sex development (DSD) include a diverse group of congenital conditions in which the development of chromosomal, gonadal, or anatomical sex is discordant, and it is classified into 3 categories: 46,XY DSD, 46,XX DSD, and sex chromosome DSD.^[1]^ Among all kinds of DSD, 46,XY DSD is one of the most common types. Most of cases of 46,XY gonadal dysgenesis result from mutations in the SRY gene. Along with SRY, mutations in NR5A1 (SF1) and MAP3K1 are the most prevalent causes of 46,XY gonadal dysgenesis.^[2]^

NR5A1 (also known as SF1) encodes an orphan nuclear receptor, which regulates the hypothalamic-pituitary-gonadal-adrenal axis. NR5A1 is crucial for normal reproductive physiology and endocrine function. Pathogenic variants in NR5A1 associated with a broad range phenotype include male infertility, hypospadias, and testicular dysgenesis in 46,XY individuals, primary ovarian insufficiency and ovotesticular DSD in 46, XX individuals.^[3]^ Here we reported a case about pathogenic variants in NR5A1 associated with 46,XY gonadal dysgenesis.

## 2. Case report

**Figure 1.**
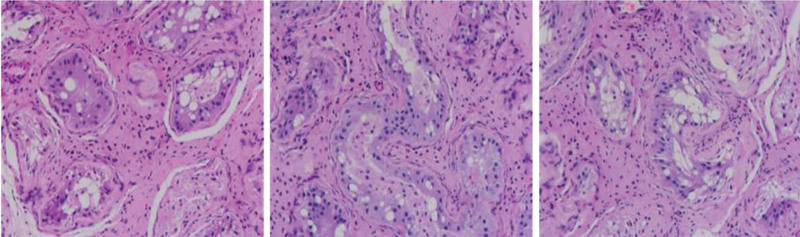
Pathological examination of the testicular biopsies. Left testes: hypoplastic seminiferous tubules seen under the microscope, consistent with the dysplastic testicular tissue. Right testes: hypoplastic seminiferous tubules seen under the microscope, consistent with the dysplastic testicular tissue. Special staining results of Ag and PAS supported the above diagnosis.

A 12-year-old individual raised as a girl was admitted to the hospital due to hirsutism and a deep voice that began at 11 years old. The individual had vellus hair on the upper lip, longer hair on the arms and legs, and had no obvious Adam’s apple. Her height was 158 cm (+1 SDS), weight 50 kg (+1 SDS), and BMI 20.0 kg/m^2^. Her blood pressure was 106/50 mmHg. She had not yet experienced menarche. Physical examination revealed clitoromegaly, with a length of 1.5 cm and width of 1.0 cm. Breast development shown Tanner stage II. She had no similar family and genetic medical history. Laboratory examination revealed the following hormone values: luteinizing hormone 17.44 mIU/mL (0.56–58.96), prolactin 14.91 ng/mL (5.12–26.91), progesterone 0.49 ng/mL (0.16–13.03), and estradiol 11.37 pg/mL (22.03–315.88); all the above values were in normal range, but follicle stimulating hormone 53.31 mIU/mL (1.78 to 11.60) and testosterone 2.58 ng/mL (0–1.23) were elevated. In addition, alpha-fetoprotein 0.83 ng/mL (0.89–8.78), insulin-like growth factor-1 377.10 ng/mL (60–350), carcinoembryonic antigen, glycobased antigen CA125, CA153, and CA199 were all negative. The cytogenetics revealed a 46,XY karyotype and DNA sequencing show a variant in NR5A1. The adrenal ultrasound show no cystic or solid mass in bilateral adrenal. The abdominopelvic ultrasound revealed hypoechoic mass (considered primordial uterus) in posterior bladder, and hypoechoic mass (considered bilateral testicles) in bilateral groin. The pelvic magnetic resonance imaging shown mixed signals in bilateral inguinal canal (considered vascular shadow). Uterus and ovaries were not visualized. The bone age DR indicated that bone age was in the normal range (12 years old). The individual^’^s clinical features, laboratory examinations, and images were consistent with 46,XY DSD with partial gonadal dysgenesis. She underwent diagnostic laparoscopy and bilateral orchiectomy at age 12 years. Pathology show testes dysgenesis, composed mainly of hypoplastic seminiferous tubules (Fig. 1). After bilateral orchiectomy, a feminizing hormonal treatment of 0.5 mg/day of estradiol valerate tablets was started, which resulted in regression of hirsutism and clitoromegaly.

## 3. Discussion and literature review

The development of gonads is unique compared to other organs as it has the potential to differentiate into testis or ovary, and the process depends on the activation of the testis-specific or ovary-specific pathway. When the pathways are disturbed, DSD happens. DSD is classified into 3 groups: (1) 46, XX DSD: comprises mainly of congenital adrenal hyperplasia, ovarian dysgenesis, and uterine/vaginal malformations; (2) Sex chromosome DSD: consists mainly of disorders with gonadal dysgenesis due to sex chromosome imbalances such as Turner syndrome (45, X and mosaicism), Klinefelter syndrome (47, XXY), mixed gonadal dysgenesis (45,X/46,XY), and chimeric DSD (46,XX/46,XY); (3) 46,XY DSD: characterized by ambiguous or female appearance of external genitalia, including hypospadias, micropenis, cryptorchidism, and testicular dysgenesis. It can be further subdivided into 3 categories: gonadal dysgenesis due to gonadal development problems, insufficient androgen production (testosterone and dihydrotestosterone) due to defects in biosynthesis, or target organ resistance to androgen due to defects in androgen receptor.^[1]^ In our case, it was the NR5A1 mutation that caused gonadal dysgenesis and resulted in 46,XY DSD.

NR5A1 is expressed in the urogenital ridge, and its gene product SF-1 upregulates the SRY expression.^[4]^ SF-1 binds to and activates testis-specific enhancer of SOX9 core (TESCO), and associates with SOX9 to regulate the anti-Müllerian hormone expression, which lead to the regression of Müllerian structures.^[5]^ NR5A1 mutations occur in gonadal and adrenal dysgenesis in individuals with 46,XY female phenotype.^[6]^ More commonly, NR5A1 mutations happening in individuals with 46,XY DSD are associated with varying severity but without adrenal insufficiency.^[7]^ NR5A1 mutations in humans was first described in a patient with 46,XY DSD, Müllerian structures, and primary adrenal failure.^[8]^ Since then, NR5A1 mutations in 46,XY DSD had been reported in different countries; 5 heterozygous NR5A1 mutations were identified in a cohort of 27 German 46,XY DSD patients with varying degrees of gonadal dysgenesis and severe androgenization.^[6]^ Two NR5A1 variations were found in a cohort of 23 Egyptian 46,XY DSD patients with hypospadias.^[9]^ Five heterozygous NR5A1 mutations were described in a cohort of 66 Brazilian patients with 46,XY DSD.^[10]^ Nineteen nucleotide mutations in NR5A1 in 25 patients were detected among 205 46,XY DSD cases in a study.^[11]^ Ten NR5A1 variations were identified among German patients with 46,XY DSD with varying phenotypes in a cohort study.^[12]^

In our case, a 12-year-old girl was diagnosed with 46,XY DSD due to NR5A1 mutation. The individual with 46,XY karyotype was noticed to have clitoromegaly, but otherwise normal female external genitalia, she has vellus hair on the upper lip, longer hair on the arms and legs, undescended dysplasia testes, no uterus, and ovaries. She underwent orchiectomy and started on hormonal replacement therapy, which resulted in the regression of clitoromegaly and hirsutism. This case highlighted the importance of considering NR5A1 variants in the diagnosis of 46,XY DSD.

## Author contributions

**Conceptualization:** Xianzhen Wei, Shan Li, Yu He.

**Data curation:** Xianzhen Wei.

**Formal analysis:** Xianzhen Wei.

**Methodology:** Shan Li, Yu He.

**Resources:** Shan Li, Yu He.

**Writing – original draft:** Xianzhen Wei.

**Writing – review & editing:** Yu He.
